# Metformin Inhibit Cervical Cancer Migration by Suppressing the FAK/Akt Signaling Pathway

**DOI:** 10.31557/APJCP.2019.20.12.3539

**Published:** 2019

**Authors:** Henna Hakimee, Pilaiwanwadee Hutamekalin, Supita Tanasawet, Pennapa Chonpathompikunlert, Varomyalin Tipmanee, Wanida Sukketsiri

**Affiliations:** 1 *Department of Pharmacology, *; 2 *Department of Physiology, *; 3 *Department of Anatomy, Faculty of Science, *; 5 *Department of Biomedical Sciences, Faculty of Medicine, Prince of Songkla University, Hat Yai, Songkhla, *; 4 *Expert Centre of Innovative Health Food (InnoFood), Thailand Institute of Scientific and Technological Research (TISTR), Pathumthani, Thailand. *

**Keywords:** Filopodia, HeLa, lamellipodia, Rac1, RhoA

## Abstract

**Background:**

Metformin, an antidiabetic drug, has been previously reported to have anti-cancer activities. However, its role in the control of cancer cell migration remains elusive.

**Methods:**

To examine the possible effect of metformin on migration of cervical cancer cells. The related mechanisms were further determined by immunocytochemistry and Western’s blotting assay.

**Results:**

The results showed that metformin treatment substantially inhibited the migration ability of cervical cancer cells. Consistently, the filopodia and lamellipodia formation were depleted after exposure to metformin. The suppression of migration mediated through the regulatory proteins such as focal adhesion kinase (FAK), ATP-dependent tyrosine kinase (Akt), Rac1 and RhoA after metformin treatment.

**Conclusion:**

Metformin displays antimigration effects in cervical cancer cells by inhibiting filopodia and lamellipodia formation through the suppression of FAK, Akt and its downstream Rac1 and RhoA protein. We propose that metformin could be a novel potential candidate as an antimetastatic cancer drug in the cervical cancer management.

## Introduction

Metastasis is the major cause of cervical cancer-related death (Thanapprapasr et al., 2010). During cell spreading, the cancer cells are able to migrate from their original sites into the nearby circulatory system. Increased of focal adhesion kinase (FAK) activity, a primary signaling pathway regulating the motility of cells, potentiates tumorigenesis and metastasis (Yoon et al., 2015). Alteration of FAK activity were likewise settled during the procurement process of metastatic cancer cells (Chen et al., 2010; Sima et al., 2013). Concerning the control of cancer cell migration, the phosphorylation of FAK at Tyr-397 are critical processes to trigger migration (Mitra et al., 2005; Lietha et al., 2007). Furthermore, the activated status of various migratory regulators such as ATP-dependent tyrosine kinase (Akt) is important for the process of cell movement (Kim et al., 2001; Huang et al., 2005). Numerous studies have demonstrated that the activation of Akt augments the efficiency of migration and invasion of cancer cells (Kim et al., 2001; Scaltriti and Baselga, 2006). Akt localizes at the edge of moving cells interacts with actin-binding proteins and induces actin remodeling and membrane protrusions formation, which subsequently promote cell motility (Kim et al., 2001). Previous studies proved the down-regulation of Akt utilizing an antisense technique and found a dramatic suppression of cancer cell invasion in vitro (Pu et al., 2004) and in vivo (Pu et al., 2006). Recently, the Rho family of small guanosine triphosphatases (GTPases), has been reported to play a crucial role in reorganization of actin and the formation of filopodia. The expression level of Rac1 and RhoA were found to be increased in several cancers including cervical cancer (Kamai et al., 2004; Liu et al., 2014). Upon the activation of Rac1 and RhoA, cancer cells migration are enlarged (Vega et al., 2008; Liu et al., 2014).

Metformin has been demonstrated to have anti-cancer activity both in vivo and in vitro (Dowling et al., 2012), and is currently being investigated the underlying mechanism. Regarding the anti-cancer properties of metformin, it is postulated both direct effects on cancer cells, specifically through abolition of the AMPK/mTOR pathway (Xiao et al., 2012). In vivo and in vitro evidences showed antiproliferative and antimigrative effects in many types of cancer including breast cancer, lung cancer, colorectal cancer, prostate cancer and ovarian cancer (Zakikhani et al., 2006; Buzzai et al., 2007; Gotlieb et al., 2008; Sahra et al., 2008). Meta-analysis of metformin found that administration of metformin was associated with a significant reduction in cancer-specific mortality in diabetes patients (Han et al., 2016). Although preclinical studies suggest possible antiproliferative effects of metformin against cervical cancer, the antimigrative mechanism of metformin use in cervical cancer remains unclear. Therefore, we aimed to investigate the possible mechanism of metformin on cancer cell migration in cervical cancer cells. 

## Materials and Methods

*Cells and Reagents*

Human cervical cancer cell lines HeLa was acquired from the American Type Culture Collection (Manassas, VA). HeLa cells were cultured in complete EMEM medium supplemented with 10% fetal bovine serum (FBS), 1% L-glutamine and 1% penicillin/streptomycin in a 5% CO2 environment at 37°C. Metformin, 3-(4,5-dimethyl-thiazol-2-yl)-2,5-diphenyl tetrazolium bromide (MTT), Hoechst 33342 and phalloidin tetramethylrhodamine B isothiocyanate were acquired from Sigma Chemical, Inc. (St. Louis, MO). Primary antibodies specific to β–actin and the secondary antibody goat anti-mouse IgG/HRP were acquired from Thermo Scientific (Waltham, Massachusetts, USA). Antibodies for Akt, p473-Akt, FAK and p397-FAK were obtained from Santa Cruz (California, USA). Rac1 and RhoA antibodies were obtained from Abcam (Cambridge, UK). 

*Cell viability and apoptosis assays*

Cell viability was assessed using the MTT assay. After the indicated treatments, the cells were added with 0.5 mg/mL of MTT at 37°C for 2 h. The MTT product was evaluated with microplate reader at 570 nm, and the percentage of viable cells was determined in relation to control. Apoptosis was determined by Hoechst 33342 staining. The cells were rinsed and incubated with 10 µg/mL Hoechst 33342 for 30 min. Fluorescence microscopy (Olympus IX51 with DP71) was used to visualize and record apoptotic cells (nuclei condensation and DNA fragmentation). 

*Cell cycle analysis*

Cell cycle alteration was investigated by flow cytometry assay using Muse cell cycle kit (Merck, MCH100106). Cells were seeded at a density of 1×10^6 ^cells/mL in 6-well plates, treated with or without metformin for 24 h. After the cells were fixed with ice cold 70% ethanol overnight, Muse® cell cycle reagent was added to the cells and incubated for 30 min at room temperature in the dark. Cell cycle distribution was then analyzed by Muse^®^ cell analyzer.

*Migration assay*

Wound healing assays were used to determine the migration activity of cervical cancer cells. For the wound healing assay, a cell monolayer was grown in a 24-well plate, and a wound space was made with a P200 pipette tip. After washing with PBS, the cells were incubated with or without metformin and allowed to migrate for 12 and 24 h. Micrographs were captured under a phase contrast microscope (Olympus DP71, Japan), and the wound spaces were determined from 6 random fields of view utilizing Olympus DP controller software. Quantitative analysis of cell migration was performed using an average wound space from 6 random fields of view, and the percentage of wound space was calculated using the following formula: % wound space  = (average space at time 0 h) - (average space at time 12 or 24 h)/(average space at time 0 h) × 100. 

*Lamellipodia and filopodia formation*

Lamellipodia and filopodia formation were investigated by a phalloidin-rhodamine staining assay. The cells were seeded at a density of 5 x 10^4^ cells/well onto a 24-well plate overnight. The cells were treated with various concentrations of metformin for 24 h. The cells were then rinsed with PBS, fixed with 4% paraformaldehyde in PBS for 10 min at 37°C, permeabilized with 0.1% Triton-X100 in PBS for 5 min, and blocked with 0.2% BSA for 1 h. Then, the cells were incubated with either 10 μg/mL of phalloidin-rhodamine in PBS for 20 min, washed 3 times with PBS. Lamellipodia and filopodia protrusion was then evaluated by fluorescent imaging (Olympus IX51 with DP71). 

*Western blotting*

Following the treatment of HeLa cells with metformin, the cells were incubated in lysis buffer containing 20 mM Tris-HCl (pH 7.5), 1% Triton X-100, 150 mM sodium chloride, 10% glycerol, 1 mM sodium orthovanadate, 50 mM sodium fluoride, 100 mM phenylmethylsulfonyl fluoride and a protease inhibitor (Sigma Chemical, St. Louis, MO) for 30 min on ice. The cell lysates were collected, and the protein content was determined using the Bradford method (Bio-Rad Laboratories, Hercules, CA). Protein from each sample (75 µg) were denatured by heating at 95°C for 5 min and subsequently loaded onto a 10% SDS-polyacrylamide gel. After separation, the proteins were transferred onto nitrocellulose membranes (Merck, Germany). The transferred membranes were blocked for 2 h in 5% nonfat dry milk in TBST (25 mM Tris-HCl (pH 7.5), 125 mM NaCl, 0.05% tween 20) and incubated with the appropriate primary antibodies at 4°C overnight. The membranes were washed three times with TBST for 15 min and incubated with horseradish peroxidase-coupled isotype-specific secondary antibodies for 1 h at room temperature. The specific protein was detected by enhancing with a chemiluminescence substrate (Supersignal West Pico; Pierce).

*Statistical analysis*

Mean data from independent experiments were normalized to the results from cells in the control group. All of the experiments were repeated at least four times. An analysis of variance (ANOVA) with a Tukey post-hoc test was conducted. A P-value of less than 0.05 was considered statistically significant.

**Figure 1:**
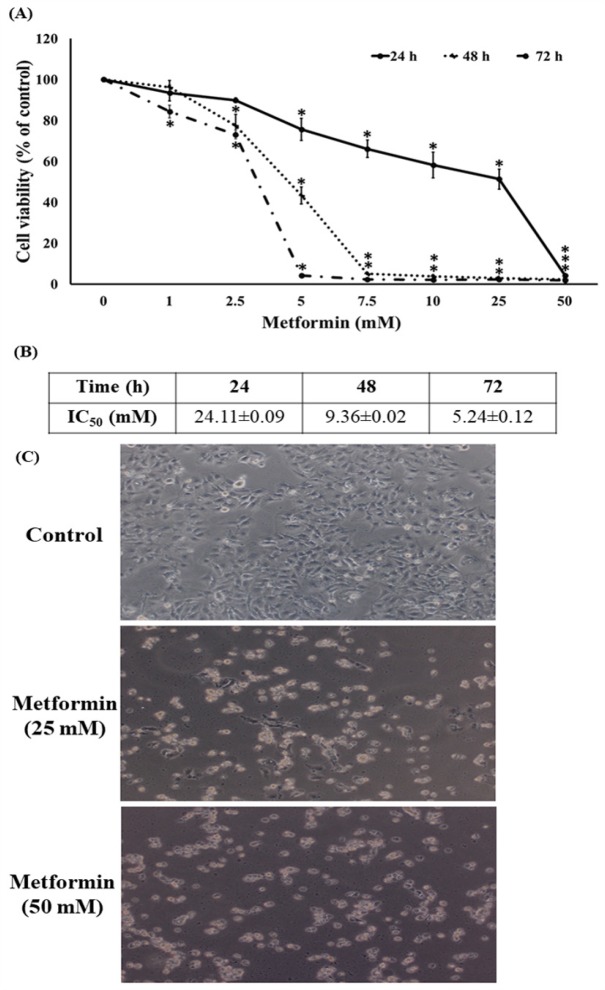
Cytotoxicity Activity of Metformin in HeLa Cells. (A) MTT assay after 24, 48 and 72 h treatment. (B) IC_50_ after 24, 48 and 72 h treatment. (C) Phase contrast microscopy (scale bar = 100 μm). Data are shown as mean ± SEM of four independent experiments. *P<0.001 versus the control

**Figure 2:**
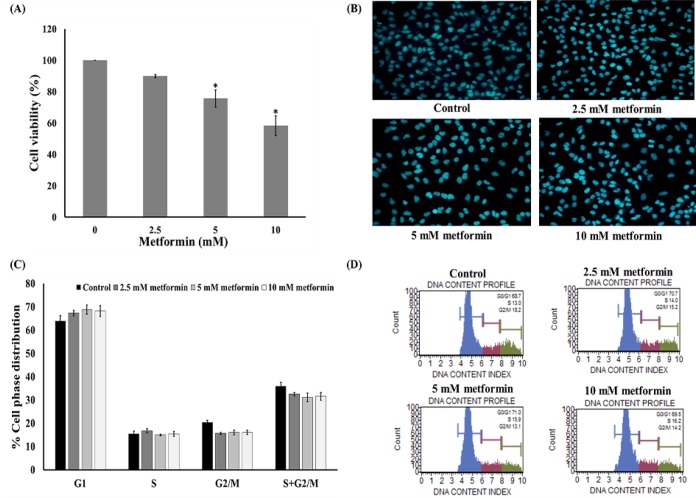
Anti-Proliferative Effect of Metformin in HeLa Cells. (A) MTT assay after 24 h treatment. (B) Hoechst 33342 staining after 24 h treatment. (C) and (D) Flow cytometry after 24 h treatment. Data are shown as mean ± SEM of four independent experiments. *P<0.001 versus the control

**Figure 3:**
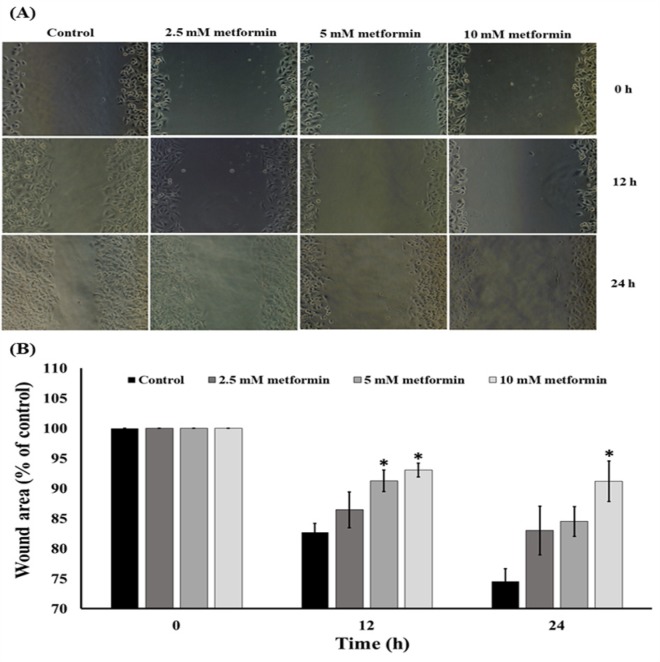
Effect of the Metformin on the Migration of Cervical Cancer Cells. (A) The cell migration was visualized via phase contrast microscopy (scale bar = 100 μm). (B) The percentage of the wound area was measured by comparing the change in wound area to that of the control. Data are expressed as mean ± SEM of four independent experiments. The difference among groups was evaluated by one-way ANOVA. *P<0.001 compared to control

**Figure 4:**
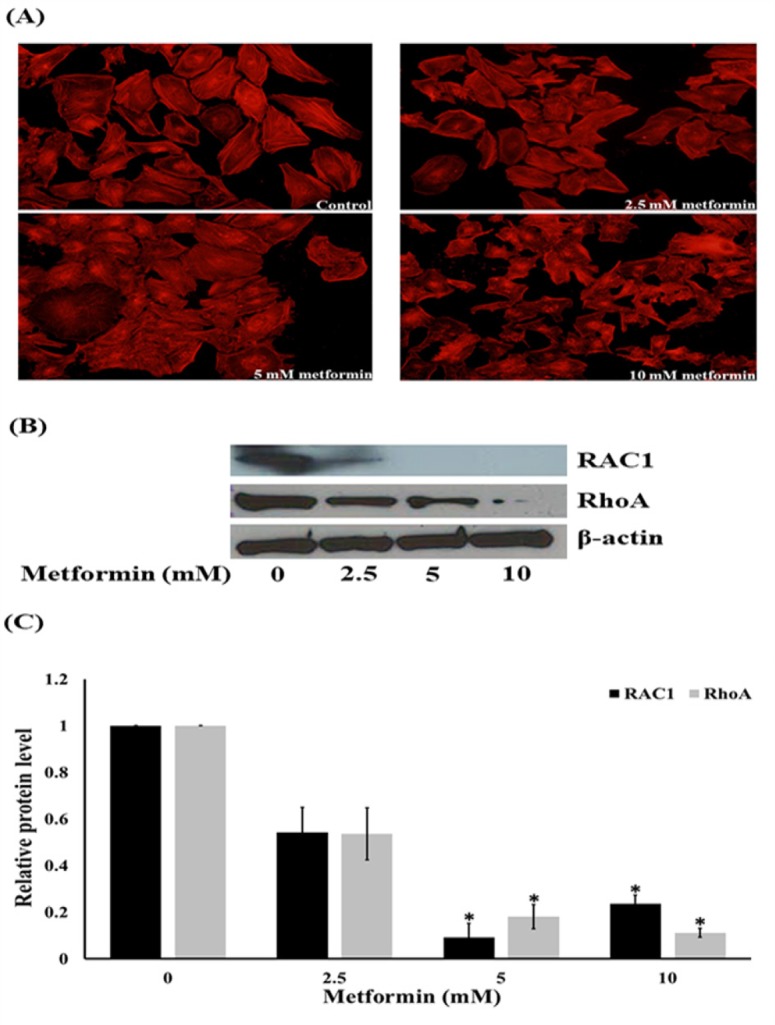
Effects of Metformin on Lamellipodia and Filopodia Formation, Ras-related C3 Botulinum Toxin Substrate 1 (Rac1) and Ras Homolog Gene Family, Member A (RhoA) Expression. (A) HeLa cells were stained with phalloidin-rhodamine (scale bar = 100 μm). (B) and (C) Western blotting and the relative level of Rac1 and RhoA in HaCaT cells after treated with metformin for 24 h. Data are shown as mean ± SEM of four independent experiments, *P<0.001 compared to control

**Figure 5:**
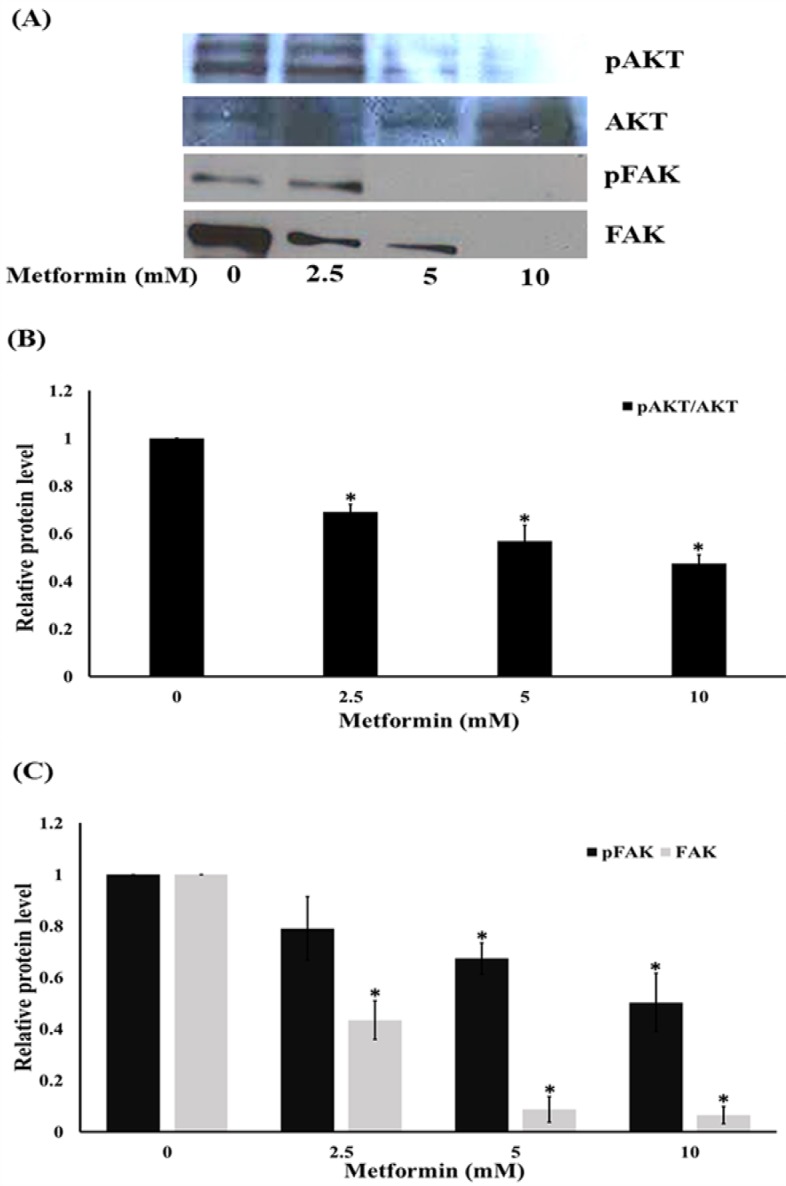
Metformin Inhibits Cell Migratory via Focal Adhesion Kinase (FAK) and ATP-Dependent Tyrosine Kinase (Akt) Activation. Western blotting (A) and the relative level of Akt (B) and FAK (C) in HeLa cells after treated with metformin for 24 h. Data are shown as mean ± SEM of four independent experiments, *P<0.001 compared to control

## Results

*Effects of metformin on cell viability*

In order to determine the cytotoxicity of metformin on human cervical cancer, HeLa cells were treated with different concentrations of metformin (0-50 mM) for 24, 48, and 72 h by MTT assay. The percentage of cell viability was decreased significantly (P < 0.001) after treatment with metformin in a dose- and time-dependent manner, and IC_50_ value of 24, 48 and 72 h time course was 24.11±0.09, 9.36±0.02 and 5.24±0.12 mM when compared to the control (Figure 1A and 1B). We further examined the cellular morphological changes of HeLa cells after treatment with the high concentration of metformin under the phase-contrast inverted microscope. We observed that metformin treatment could reduce the number of viable cells accompanied by cell rounding and detachment of cells from the tissue culture plate when compare to the control (Figure 1C). 

*Effects of metformin on cell proliferation*

Cytotoxicity experiment was performed to test the effect of metformin on HeLa cell viability. The cells were cultured in the absence or presence of metformin (2.5-10 mM) and the viability of the cells was determined using an MTT assay after treatment for 24 h. The results indicated that low concentrations of metformin (2.5 mM) caused neither toxic nor proliferative effects on the cervical cancer cells (Figure 2A). In contrast, high concentration of metformin (5-10 mM) caused a significant reduction of HeLa cell proliferation (Figure 2A). Additionally, the concentrations of metformin in the range of 0-10 mM did not induce apoptosis as detected by a Hoechst 33342 nuclear staining assay (Figure 2B). However, the cells were treated with 0-10 mM metformin for 24 h and then the cells were subjected to flow cytometry. Figure 2C and 2D showed that treatment of cells with 2.5-10 mM metformin caused no significant alteration of the cell cycle in comparison to the untreated control cells. 

*Metformin inhibits cervical cancer migration*

Low concentrations (0-10 mM) of metformin were further tested for their possible effect on cancer cell migration. The cells were treated with 0-10 mM metformin for 12 and 24 h. A wound migration assay revealed that metformin suppressed HeLa cell migration in a dose-dependent manner compared with the untreated control cells (Figure 3A and 3B). 

*Metformin reduces filopodia and lamellipodia formation in HeLa cells*

Regarding the effect of metformin on the inhibition of HeLa cells, we further investigated whether metformin affect the formation of lamellipodia and filopodia in HeLa cells. The cells were cultured in the presence or absence of metformin (0-10 mM) for 24 h, the lamellipodia formation and filopodia protrusion were then photographed under fluorescence modes of a microscope using phalloidin assays. Figure 4A showed that HeLa cells in the moving state exhibited a substantial number of filopodia protrusions and lamellipodia formation that were dramatically reduced in response to metformin treatments. The reduction of lamellipodia and filopodia in the cells is probably due to the negative regulatory role of metformin. Because the expression level of Rac1 and RhoA was shown to be tightly correlated with the filopodia and lamellipodia formation, we further investigated the mechanism of metformin in the control of filopodia and lamellipodia in HeLa cells by determining the cellular expression of Rac1 and RhoA. The results indicated that the expression level of Rac1 and RhoA dramatically decreased in response to metformin treatments (Figure 4B and 4C). This information indicates that metformin may reduce the migratory activity of HeLa cells via Rac1 and RhoA attenuation.

*Metformin attenuates activation of FAK and Akt signaling pathway*

To elucidate the mechanisms of metformin inhibited cancer cell motility, the expression and activation levels of proteins involved in cell migration were evaluated. The cells were treated with low concentrations of metformin or without metformin for 24 h. Western blot analysis of Akt, activated Akt (phosphorylated Akt at Ser473), FAK, and activated FAK (phosphorylated FAK at Tyr-397) were determined. Figure 5 showed that treatment of cells with metformin (0-10 mM) substantially down-regulated the expression of activated Akt (Figure 5A and 5B) and activated FAK while the non-phosphorylated forms of FAK proteins were also decreased (Figure 5A and 5C). These results suggest that metformin interfered with both the activated and non-activated level of the proteins and possibly regulated cell migration by inhibiting Akt and FAK downstream pathways. 

## Discussion

Normal cell migration plays a crucial role in various physiological processes, including embryonic development, tissue repair and regeneration, however it also encourages the pathological progression of cancer metastasis (Thiery et al., 2009). In cervical cancer, higher lethality became higher metastasis observed. Thus, the therapeutic regimens which markedly abolish the metastasis of cancer cells become of clinical importance. Metformin, a glucose lowering drug with a potential in anti-cancer activity, has been reported to diminish the incidence and progression of multiple human cancers (Wurth et al., 2013; Janzer et al., 2014), and improve overall survival rate of cancer patients (Currie et al., 2012). Previous studies demonstrated that metformin suppressed cervical cancer cell proliferation, migration, invasion and enhanced apoptosis (Rattan et al., 2011; Xiao et al., 2012; Cerezo et al., 2013). Nevertheless, the mode of metformin inhibiting cervical cancer metastasis is yet to be pinpointed. Herein, we demonstrated that low concentrations of metformin contribute to suppression of the migration behavior of cervical cancer cell and migratory related-protein. 

Growing evidence both from in vitro and in vivo studies indicated metformin had the direct effect on many types of cancer cells with an IC_50_ value in a range of 25-50 mM (Heckman-Stoddard et al., 2016). These findings are consistent with our results shown in Figure 1. Additionally, metformin can suppresses PI3K/Akt/mTOR signal pathway and has been presented to have chemopreventive activities against cervical cancer. Metformin is currently being analyzed as a therapeutic alternative with insulin-dependent and insulin-independent mechanism of action against a several of cancer types (Gong et al., 2016). Previous study also reported that metformin inhibits migration in cervical cancer cells (Xia et al., 2017). Consistent with a previous finding (Xia et al., 2017), our results demonstrated that metformin inhibits the ability of cervical cancer cells to migrate. The cancer cell migration implicates various cellular signals and most of them are associated with the invasive function of the cells. Meanwhile, the specific mechanisms are still being interrogated, various proteins such as focal adhesion kinase (FAK) and Akt have been shown to control cancer cells motility. Tyrosine kinases, such as FAK, have been displayed to play an essential role in cell motility via signal communication to the downstream cellular machinery through their activity of kinase (Hsia et al., 2003). Phosphorylation of FAK at Tyr-397 is a requisite for its activated state, and phosphorylated FAK was increasingly exhibited in highly motile and invasive cancer cells (Miyazaki et al., 2003). In the present study, we determined both total and phosphorylated FAK reductions in metformin-treated cells. Even though abundant studies support the function of the mentioned proteins individually and the pathways modulating the motility of cancer cell are quietly and broadly unknown, a consequential number of research works have examined their regulatory impact on each other. Stimulated FAK was displayed to activate Akt, and these stimulations are required in the migration of cell (Meng et al., 2009). Based on our data, it is possible that metformin may restrain the activation of FAK and downregulate function of Akt via its downstream effectors (Hsia et al., 2003; Meng et al., 2009). Specifically, the phosphorylation of Akt at Ser473 was shown to be important for the migration of cancer cell (Shukla et al., 2007; Zhau et al., 2011). The phosphorylated FAK and Akt trigger its downstream effectors including Rho families (Zhao et al., 2011). The Rac1 and RhoA-GTP downstream migratory protein controls the migration of cell by activating the development of cell-membrane protrusion filopodia at the boundary of the migrating side (Vega and Ridley, 2008; Liu et al., 2014). During the movement of cells, filopodia and lamellipodia increase and are required in migration and metastasis of cancer (Machesky, 2008; Dirat et al., 2014). In this study, we found that the reduction of Rac1 and RhoA in response to metformin treatment is in association with the reduction of cellular filopodia and lamellipodia.

In conclusion, metformin inhibits migration with possible modes of action covering deterioration of migratory-correlated Rac1 and RhoA, and abolition of FAK/Akt signals which subsequently inhibited the downstream migratory proteins. Our finding serves as a novel preliminary alternative of metformin application as a promising anti-metastatic agent in cervical cancer treatment.
